# Overexpression of TGR5 alleviates myocardial ischemia/reperfusion injury via AKT/GSK-3β mediated inflammation and mitochondrial pathway

**DOI:** 10.1042/BSR20193482

**Published:** 2020-01-24

**Authors:** Junzhi Li, Ruining Cheng, Hong Wan

**Affiliations:** 1 Department of Emergency, The Ninth Hospital of Xi’an, Xi’an 710054, Shaanxi, China; 2 The First Department of Geriatrics, The Ninth Hospital of Xi’an, Xi’an 710054, Shaanxi, China; 3 Department of Emergency, The TCM Encephalopathy Hospital of Xi’an, Xi’an 710032, Shaanxi, China

**Keywords:** AKT/GSK-3β, apoptosis, inflammation, schemia/reperfusion (I/R), TGR5

## Abstract

Ischemia/reperfusion (I/R) injury reduces cell proliferation, triggers inflammation, promotes cell apoptosis and necrosis, which are the leading reasons of morbidity and mortality in patients with cardiac disease. TGR5 is shown to express in hearts, but its functional role in I/R-induced myocardial injury is unclear. In the present study, we aimed to explore the underlying molecular mechanism of TGR5 in hypoxia/reoxygenation (H/R)-induced cardiomyocyte injury *in vitro*. The results showed that TGR5 was significantly up-regulated in H9C2 (rat cardiomyocyte cells) and human cardiomyocytes (HCMs) after H/R. Overexpression of TGR5 significantly improved cell proliferation, alleviated apoptosis rate, the activities of caspase-3, cleaved caspases-3 and Bax protein expression levels, and increased Bcl-2 level. Overexpression of TGR5 significantly up-regulated ROS generation, stabilized the mitochondrial membrane potential (MMP), and reduced the concentration of intracellular Ca^2+^ as well as cytosolic translocation of mitochondrial cytochrome *c* (cyto*-c*). Meanwhile, overexpressed TGR5 also enhanced the mRNA and protein levels of interleukin (IL)-10, and decreased the mRNA and protein levels of IL-6 and tumor necrosis factor α (TNF-α). The shTGR5+H/R group followed opposite trends. In addition, overexpressed TGR5 induced an increase in the levels of p-AKT and p-GSK-3β. The protective effects of TGR5 were partially reversed by AKT inhibitor MK-2206. Taken together, these results suggest that TGR5 attenuates I/R-induced mitochondrial dysfunction and cell apoptosis as well as inflammation, and these protections may through AKT/GSK-3β pathway.

## Introduction

Myocardial ischemia/reperfusion (I/R) injury causes extreme reactions after myocardial ischemia, cardiac surgery or circulatory arrest and is one of the main causes of the morbidity and mortality in patients with cardiac disease such as myocardial infarction and coronary heart disease [1,2]. The mounting evidence suggested that myocardial I/R leads to autophagy and apoptosis through the activation of caspase-cascade by a release of cytochrome *c* (cyto-*c*) from mitochondria to cytoplasm, and triggers an inflammatory response and releases inflammatory cytokines via activating inflammatory related pathway [3–5]. However, the underlying molecular mechanisms of myocardial I/R-induced apoptosis and mitochondria dysfunction as well as inflammation are not fully understood.

G protein-coupled receptor TGR5, also named as GPBAR1, BG37 or M-BAR, belonged to G-protein-coupled receptors (GPCRs) family [6]. TGR5 participates in the regulation of multiple cellular signaling pathways such as AKT pathway, and its agonist may be potential drugs for treatment of inflammation, metabolic and digestive disorders [7,8]. In addition, TGR5 promotes mitochondrial fission and beige remodeling of white adipose tissue [9]. Desai et al. (2010) [12] first reported that TGR5 was expressed in cardiomyocytes [10]. In recent years, studies have shown that TGR5 plays a key role in myocardial adaptability, and activated TGR5 may be a potentially attractive treatment option in cardiac hypertrophy and heart failure [11,12]. At present, reports indicated that that TGR5 alleviates the liver I/R-related inflammation and protects hepatocytes from I/R-related apoptosis [13,14]. However, the role and function of TGR5 in myocardial I/R injury remained poorly understood.

The present study examined the putative role of TGR5 in the pathophysiology of the TGR5 or shTGR5 cardiac warm hypoxia/reoxygenation (H/R) injury model. Our results demonstrated that TGR5 can ameliorate mitochondrial dysfunction, cell apoptosis and inflammation due to H/R, suggesting the protective effects of TGR5 in the maintenance of myocardial homeostasis via the activation of the AKT/GSK-3β signaling pathway.

## Materials and methods

### Cell culture and building I/R model

H9C2 and human cardiomyocyte (HCM) cells were cultured in Dulbecco’s Modified Eagle’s Medium (DMEM, Sigma, U.S.A.), which is composed of 10% fetal bovine serum and 100 U/ml penicillin. Then, the culture was placed in an incubator containing 5% CO_2_ at 37°C. To stimulate hypoxia, cells were incubated in a hypoxic incubator containing 95% N_2_ and 5% CO_2_ for 4 h at 37°C. Subsequently, reoxygenation (95% air, 5% CO_2_, 37°C) was performed for another 4, 6, and 12 h. Cells were collected after the ending reoxygenation. The control cells were cultured under normoxic conditions.

### Transfection of overexpression and interference plasmids

Overexpression plasmid of TGR5 was constructed and purchased from Bio‐transduction Lab (Wuhan, China). Lentivirus‐mediated short hairpin RNA (shRNA) targeting TGR5 mRNA was synthesized by Shanghai GeneChem (Shanghai, China). Overexpression plasmid of TGR5 and TGR5 shRNA (shTGR5) were transfected into cells using Lipofectamine 2000 transfection reagent (Thermo Fisher Scientific, Wilmington, DE), according to the instructions.

### Extraction RNA and RT-qPCR

Total RNA was extracted using RNAiso Plus Reagent (TaKaRa, Dalian, China) and NanoDrop 1000 Spectrophotometer (Thermo Scientific; Madrid, Spain) was used to check the quality and concentration of RNA. The first strand complementary DNA (cDNA) were synthesized by using PrimeScript® RT Reagent Kit (TaKaRa, Dalian, China). The expression of TGR5, interleukin (IL) 10 (IL-10), IL-6 and tumor necrosis factor α (TNF-α) was quantified using SYBR Premix Ex Taq (TaKaRa, Dalian, P.R. China) and β-actin was used as the internal control. The primers for TGR5, IL-10, IL-6, TNF-α and β-actin are as follows:

TGR5 F: 5′-CAGTCTTGGCCTATGAGCGT-3′;

TGR5 R: 5′-CTGCCCAATGAGATGAGCGA-3′;

IL10 F: 5′-ACTGCACCCACTTCCCAGT-3′;

IL10 R: 5′-TGTCCAGCTGGTCCTTTGTT-3′;

IL6 F: 5′-GCTACCAAACTGGATATAATCAGG A-3′;

IL6 R: 5′-CCAGGTAGCTATGGTACTCCAGAA-3′;

TNF-α F: 5′-GCCTCTTCTCATTCCTGCTTGT-3′;

TNF-α R: 5′-TTG AGA TCC ATG CCGTTG-3′;

β-actin F: 5′-CTAAGGCCAACCGTGAAAAG-3′;

β-actin R: 5′-GCCTGGATGGCTACGTACA-3′.

RT-PCR was performed on the ABI 7300 Sequence Detection System (Applied Biosystems, Foster City, CA, U.S.A.). The relative gene expression was calculated by the 2^−ΔΔ*C*_T_^ method.

### Proliferation assay

H9C2 and HCM cells were distributed into a 12-well plate (density 1 × 10^5^ cells/well). Cell proliferation was evaluated by the Cell Counting Kit-8 (CCK8) (Dojindo Molecular Technologies, Shanghai, China) according to the instructions.

### ROS content detection

H9C2 and HCM cells were planted into 12-well plates at a density of 1 × 10^5^ cells/well. ROS content was detected through Reactive Oxygen Species Assay Kit (Beyotime Biotechnology, Shanghai, China) according to the instructions.

### Determination of intracellular calcium concentration

The intracellular calcium concentration was detected by Fluo-3 AM (Beyotime Institute of Biotechnology, Haimen, China), following the instructions.

### Mitochondrial membrane potential assay

Mitochondrial membrane potential (MMP) was detected using a mitochondrial membrane potential assay kit with JC-1 (Beyotime Institute of Biotechnology, Haimen, China) according to the manufacturer’s protocol.

### Cyto-*c* detection

Cell Mitochondria Isolation Kit (Abcam, ab110170, Cambridge, U.K.) was used to isolate mitochondria and cytosol, according to the instructions. Sample protein of the cyto-*c* subjected to Western blotting.

### Apoptosis test

Cell apoptosis analysis was performed using the Annexin V-FITC Apoptosis Detection Kit (Beyotime), according to the instructions (Beckman Coulter, Brea, CA).

### Western blot assay

The cells were collected and lysed using RIPA buffer (Biocolors Biotechnology Co., Shanghai, China). The protein was obtained by centrifuging (1000×***g***, 10 min) at low temperature and then using BCA Protein Assay Kit (Pierce; Thermo Fisher Scientific, Inc.) to ensure the concentration. Proteins were separated on 10–12% SDS/PAGE gels and transferred to polyvinylidene fluoride (PVDF) membranes. After being blocked with 5% non-fat milk with 1 h, primary antibody against TGR5, cyto-*c*, Bax, Bcl-2, cleaved caspases-3, p65, Lamin B1, p-IκB-α, IκB-α, p-AKT, p-GSK-3β and β-actin (Cell Signaling Technology, Inc.) were incubated overnight at 4°C. Finally, the membranes were incubated with horseradish peroxidase–conjugated secondary antibodies for 1 h at room temperature. β-actin was used as an internal control for the correction of protein expression. Finally, the protein bands were detected using an enhanced chemiluminescence detection system (Thermo Fisher Scientific, Inc.) and the relative band densities were quantified using the Multi-Analyst software package (Bio-Rad).

### Enzyme-linked immunosorbent assay

The activity of caspase-3 was assayed by using Caspase 3 Activity Assay kit (Beyotime Biotechnology, Shanghai, China), according to the instructions. And serum expression of IL-10, IL-6 and TNF-α were examined using commercial kits according to protocols recommended by the manufacturers (IL-10: RayBiotech Inc, Norcross, GA, U.S.A.; IL-6 and TNF-α: BIOTREND Chemikalien GmbH, Koln, Germany).

### Statistical analysis

SPSS 22.0 was used to analyze the experimental data. The significance was analyzed by Student’s *t* test or one-way ANOVA. The data were represented as the mean ± SD. All experiments have three biological repetitions where each one has three technological repetitions. *P*<0.05 was considered with statistically significant.

## Results

### TGR5 is induced by H/R in H9C2 and HCM cells

**Figure 1:**
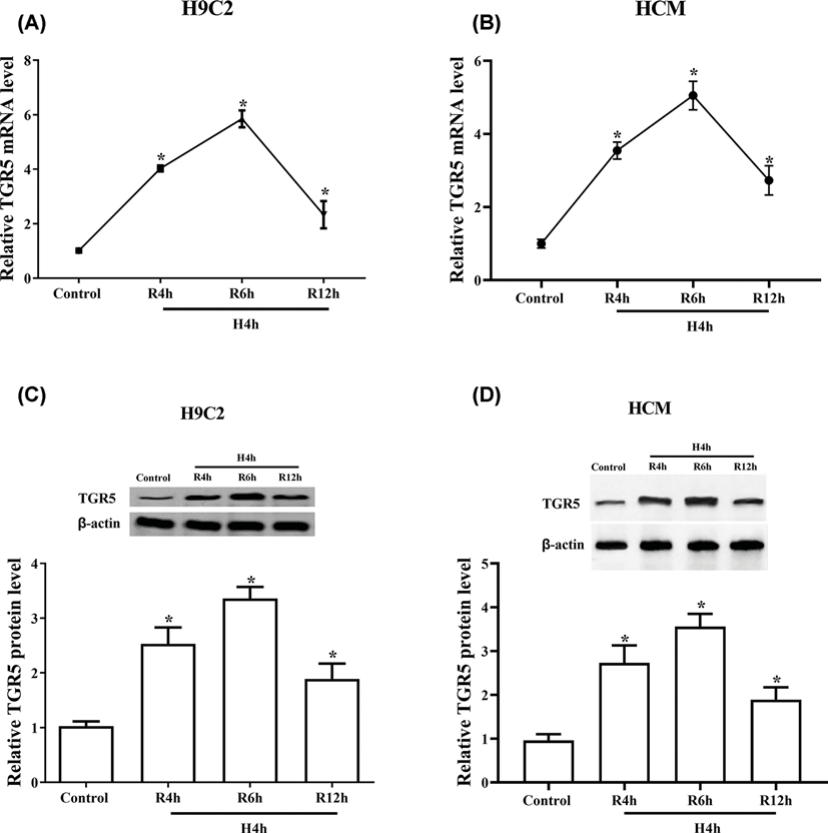
The expression of TGR5 was induced by H/R in H9C2 and HCM cells Cells were exposed in hypoxic condition for 4 h followed by 4, 6 and 12 h of reoxygenation. The mRNA level of TGR5 was measured in H9C2 and HCM cells by RT-qPCR (**A,B**). The protein level of TGR5 was checked by Western blot in H9C2 and HCM cells (**C,D**). *P<0.05 vs control group; *n*=3, N=3.

TGR5 gene expression was evaluated using RT-qPCR and Western blot assays. Results indicated that the mRNA level of TGR5 was significantly increased following reoxygenation for 4, 6 and 12 h in H9C2 and HCM cells (*P*<0.05), with a peak at 6 h and then falling rapidly (Figure 1A and B). The trend of the protein level of TGR5 is similar with mRNA level of TGR5 and the expression of TGR5 protein was also up-regulated in H9C2 and HCM cells (*P*<0.05) (Figure 1C and D). Those results showed that the level of TGR5 can be induced by H/R injury in H9C2 and HCM cells.

### Overexpression of TGR5 decreases H/R-induced cell apoptosis in H9C2 and HCM cells

**Figure 2:**
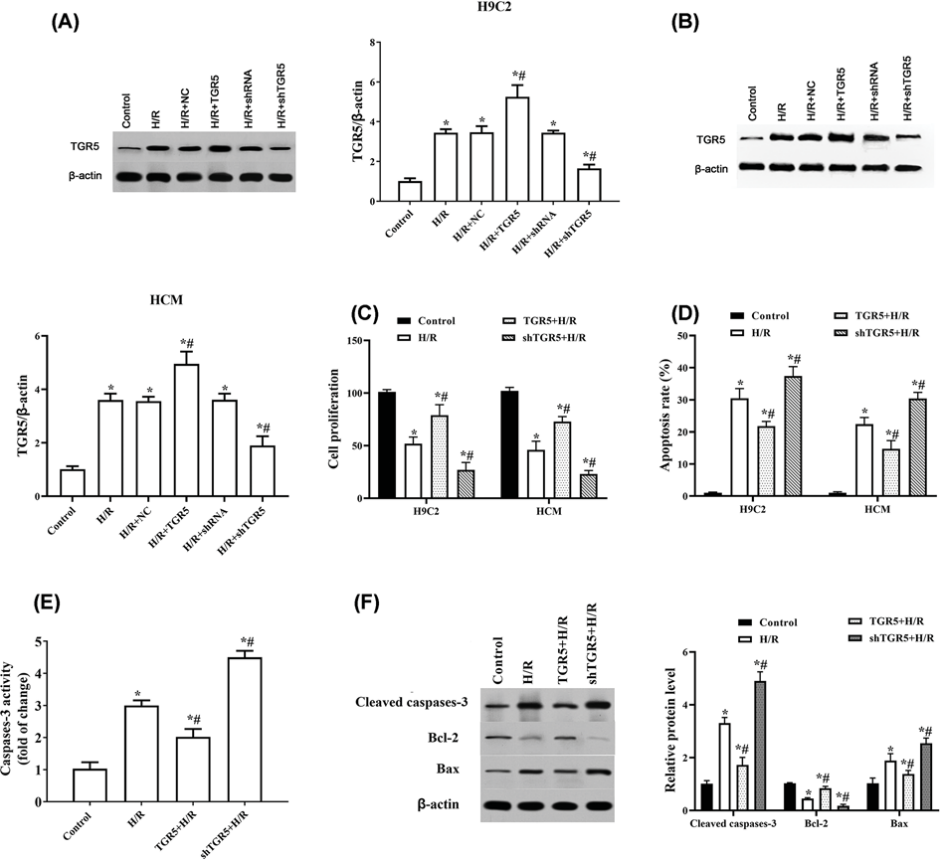
TGR5 regulates H/R-induced apoptosis in H9C2 and HCM cells Cells were exposed in hypoxic condition for 4 h followed by 6 h of reoxygenation then transfected with pcDNA3.1-TRG5 or Lentivirus-mediated TRG5-shRNAs (**A**). The transfection effect of pcDNA3.1-TRG5 or Lentivirus-mediated TRG5-shRNAs was checked by Western blot in H9C2 and HCM cells. NC: empty plasmids; TRG5: pcDNA3.1-TRG5; shRNA: control-shRNA (**B**). Cell proliferation was examined by CKK8 in H9C2 and HCM cells (**C**). Apoptosis rate was examined by flow cytometry assay in H9C2 and HCM cells (**D**). Caspases3 activity was measured through ELISA in H9C2 cells (**E**). The level of cleaved-caspases-3, Bcl-2 and Bax protein were checked by Western blot in H9C2 cells (**F**). H/R: Hypoxia for 4 h and reoxygenation for 6 h; *P<0.05, compared with control group; ^#^*P*<0.05, compared with H/R group; *n*=3, N=3.

To determine the functional significance of TGR5 in H/R injury, gain- and loss-of-function approaches were employed in cultured H9C2 and HCM cells by transfection of overexpression and interference plasmids. With the up-regulation of TGR5 by transfection of pcDNA3.1‐TRG5, cell proliferation was significantly increased compared with I/R group, but the shTGR5+H/R groups significantly reduced proliferation compared with those in the H/R group in H9C2 and HCM cells (Figure 2A-C). In addition, the apoptosis rate was down-regulated after overexpression of TGR5 and increased by transfecting shTGR5, compared with H/R group in H9C2 and HCM cells (*P*<0.05, Figure 2D). Furthermore, the activity of caspases-3, the pro-apoptosis protein of cleaved-caspases-3 and Bax were down-regulated after overexpression of TGR5 and increased by transfecting shTGR5, compared with H/R group in H9C2 cells (*P*<0.05) (Figure 2D–F). Consistent with this, the level of anti-apoptosis protein Bcl-2 was up-regulated by TGR5 overexpression and down-regulated by TGR5 inhibition in H9C2 cells (Figure 2F). These data indicated that TGR5 significantly regulates H/R-induce apoptosis.

### TGR5 regulates mitochondrial pathway in H9C2 and HCM cells

**Figure 3:**
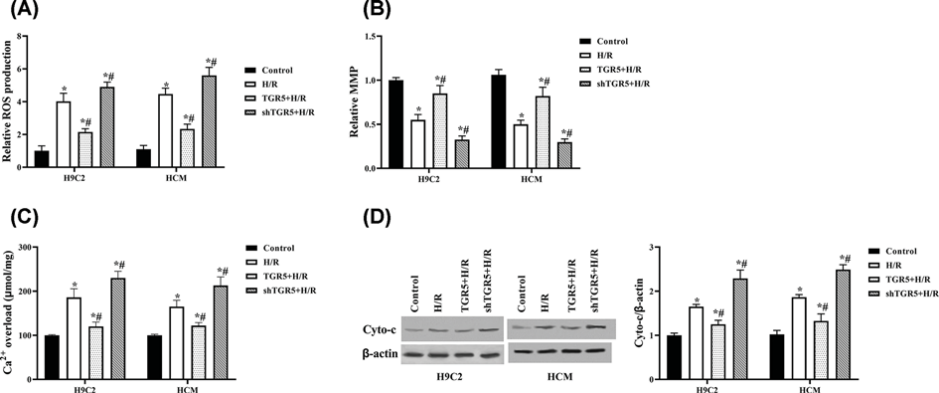
Effect of overexpression and knockdown of TGR5 on H/R-induced mitochondrial pathway in H9C2 and HCM cells Cells were placed in hypoxic condition for 4 h followed by 6 h of reoxygenation then transfected with pcDNA3.1-TRG5 or Lentivirus-mediated TRG5-shRNAs. The level of intracellular ROS was examined by Reactive Oxygen Species Assay Kit (**A**). MMP was detected by fluorescent dye JC-1 (**B**). Calcium concentration was measured through Fluo-3 AM (**C**). The release of Cyto-c was detected by Western blot (**D**). H/R: Hypoxia for 4 h and reoxygenation for 6 h; *P<0.05, compared with control group; ^#^*P*<0.05, compared with H/R group; *n*=3, N=3.

Mitochondrial functional and structural integrity play central roles in regulating the apoptotic process. Therefore, we guessed that the effects of TGR5 inhibiting apoptosis may be associated with mitochondria dysfunction. As shown in Figure 3A,C,D, the relative level of ROS, the concentration of intracellular Ca^2+^ and the relative expression of cyto-*c* were down-regulated in TGR5+ H/R group and up-regulated in shTGR5+ H/R group, compared with H/R group in H9C2 and HCM cells (*P*<0.05). However, MMP was improved in TGR5+ H/R group and alleviated in shTGR5+ H/R group, compared with H/R group in H9C2 and HCM cells (Figure 3B). The above results proved that the anti-apoptosis effects of TGR5 may be related with mitochondria dysfunction.

### Overexpression of TGR5 alleviates H/R-induced inflammation in H9C2 and HCM cells

**Figure 4:**
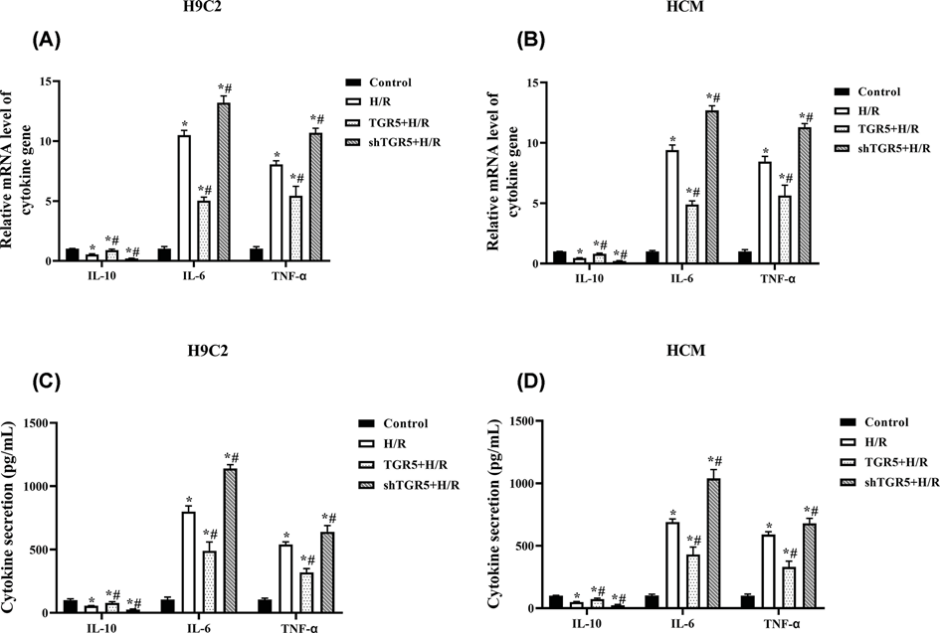
TGR5 regulates inflammatory response in H/R-stressed cardiomyocytes H9C2 and HCM cells were exposed in hypoxic condition for 4 h followed by 6 h of reoxygenation then transfected with pcDNA3.1-TRG5 or Lentivirus‐mediated TRG5-shRNAs. (**A and B**) Cytokine genes (*IL-10, IL-6* and *TNF-α*) expression were measured through RT-qPCR analysis. (**C and D**) ELISA was used to analyze cytokine secretions (IL-10, IL-6 and TNF-α) H/R: Hypoxia for 4 h and reoxygenation for 6 h; *P<0.05, compared with control group; ^#^*P*<0.05, compared with H/R group; *n*=3, N = 3. Abbreviation: ELISA, enzyme-linked immunosorbent assay

Anti-inflammatory cytokine (IL-10) exhibits a protective role in I/R-stressed cardiomyocytes. However, inflammatory cytokines (TNF-α and IL-6) display pro-inflammatory effect in ischemia cardiomyocytes after reperfusion. To further ensure the cardioprotective effects of TGR5, the transcriptional levels of IL-10, IL-6 and TNF-α were determined (Figure 4A and B). Compared with H/R groups, TGR5+ H/R group increases level of IL-10, and reduces levels of TNF-α and IL-6 in H9C2 and HCM cells (*P*<0.05). However, shTGR5+ H/R group significantly down-regulates level of IL-10 and improves levels of TNF-α and IL-6, compared with H/R groups in H9C2 and HCM cells (*P*<0.05). Similar results were found in serum levels of IL-10, IL-6 and TNF-α (*P*<0.05) (Figure 4C and D). Furthermore, we revealed that overexpression of TGR5 inhibited the phosphorylation of IκBα and p65 translocation that induced by H/R, and the phosphorylation of IκBα and p65 translocation were further increased in shTGR5+ H/R group compared with H/R group in H9C2 cells (Supplementary Figure S1).

### Overexpression of TGR5 alleviates inflammation and mitochondrial pathway via activating AKT/GSK-3β pathway in H9C2 cells

**Figure 5:**
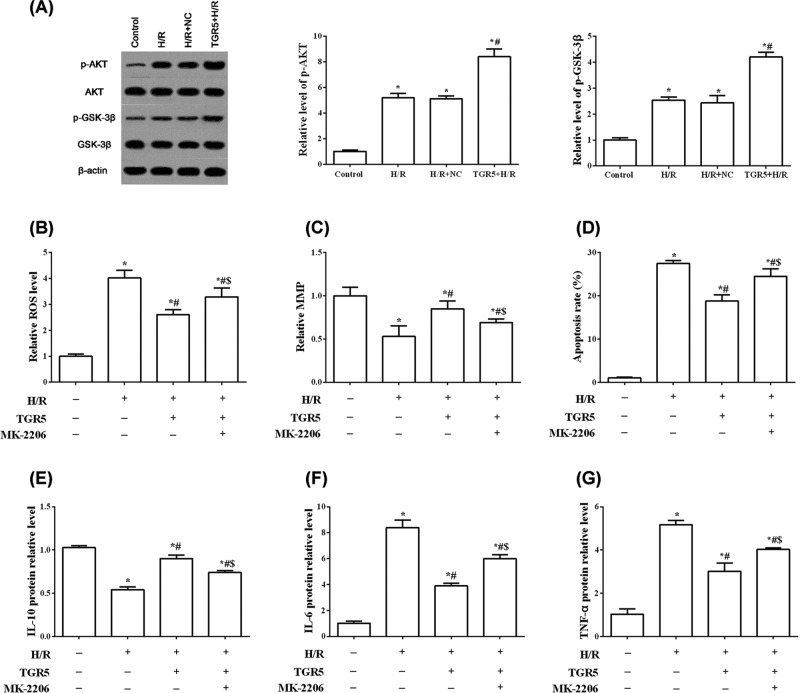
TGR5 participates in the regulation of AKT/GSK-3β pathway in H9C2 cells (**A**) The levels of p-AKT and p-GSK-3β were measured through western blot. (**B**) The level of intracellular ROS, (**C**) mitochondrial membrane potential (MMP) and (**D**) apoptosis rate were measured with fluorescent dye CM-H2DCFDA, JC-1, and flow cytometry. The protein levels of IL-10(**E**), IL-6 (**F**) and TNF-α (**G**) were also checked by western blot. H/R: Hypoxia for 4h and reoxygenation for 6h; *P<0.05, compared with control group; ^#^*P*<0.05, compared with H/R group; ^$^*P*<0.05, compared with TGR5+H/R group; *n*=3, N=3.

To gain a comprehensive understanding the mechanism of TGR5 in regulating myocardial H/R-induced apoptosis, mitochondrial pathway and inflammation, we further examined the AKT/GSK-3β pathways. Western blot analysis showed that levels of phosphorylated AKT and GSK-3β were significantly increased by overexpression TGR5 (*P*<0.05) (Figure 5A). The specific inhibitor of AKT (MK-2206) partially abolishes the protective effects of overexpression of TGR5 against H/R-induced H9C2 impairment (Figure 5B–G). Treatment of MK-2206 improves the level of ROS and apoptosis rate and alleviates MMP, compared with TGR5+H/R group (*P*<0.05) (Figure 5B–D). Consistently, MK-2206 increases the levels of IL-6 and TNF-α and reduces the level of IL-10, compared with TGR5+H/R group (*P*<0.05) (Figure 5E–G**)**. These data suggested that overexpressed TGR5 alleviates mitochondrial pathway and inflammation via the activation of AKT/GSK-3β pathway.

## Discussion

Here, we studied the function and mechanism of TGR5 in H/R-induced injury in H9C2 and HCM cells. We found that the levels of TGR5 mRNA and protein were significantly increased and peaked after 6 h of reperfusion following Hypoxia for 4 h, which is similar to the studies of Yang et al. [13] and Zhuang et al. [14] who reported that the level of TGR5 improved in the early stages of reperfusion and peaked at 6 h after reperfusion in the liver and hepatocytes after I/R injury. Besides, our present results demonstrated that overexpressed TRG5 alleviates myocardial I/R-induced apoptosis rate and regulates the levels of apoptosis-related proteins. Plentiful experiments suggest that myocardial I/R leads to cell death, which is thought to occur through necrosis and apoptosis. Kajstura et al. [15] showed that apoptosis was the predominant mode of cardiac cell death induced by coronary artery occlusion [16]. These results revealed that overexpressed TRG5 protects cardiomyocytes from apoptosis induced by H/R.

Previous studies showed that myocardial I/R induces mitochondrial dysfunction including the enhancement of ROS generation, mitochondrial Ca^2+^ overload, the depolarization of MMP and the releases of the proapoptotic protein, cyto-*c*, from the mitochondria [17–19]. The release of cyto-*c* from the mitochondria to cytoplasm caused by the change of mitochondrial membrane permeability is the key link of apoptosis induced by intracellular mitochondrial pathway [20]. Therefore, mitochondrial functional and structural integrity perform a central role in regulating the apoptotic process [18]. Velazquez-Villegas et al. [9] reported that overexpressed TGR5 can increase mitochondrial content, induce mitochondrial fission through the ERK/DRP1 pathway, further improving mitochondrial respiration. It has been reported previously TGR5 signaling pathway activates mitochondrial oxidative phosphorylation and increases the ATP/ADP ratio in enteroendocrine cells [21]. Here, we demonstrated that overexpression of TGR5 plays a positive regulation in H/R-induced mitochondrial dysfunction. These data indicated that TGR5 protects H/R-induced mitochondrial dysfunction.

Inflammatory response plays a pathogenic role in myocardial I/R injury, especially innate immune responses involved in cytokines, including IL-10, IL-6 and TNF-α, and so on [22–24]. In our study, overexpressed TGR5 increases the level of IL-10 and decreases the levels of IL-6 and TNF-α. This is consistent with other studies that TGR5 overexpression had significantly higher levels of IL-10, yet lower levels of TNF-α and IL-6 in liver and hepatocytes after I/R injury [13,14]. In addition, Pols et al. [25] reported previously that protein level of TNF-α was higher in the Tgr5^−/−^ compared with Tgr5^+/+^ mice upon LPS stimulation, and LPS-INT-777 treatment significantly decreases the mRNA levels of IL-6 and TNF-α in primary macrophages. The activation of NF-κB is one of the critical cellular responses to inflammations [26]. Previous reports demonstrated that TGR5 overexpression inhibited the activation of NF-κB signaling pathway through suppressing IκBα phosphorylation and p65 translocation in inflammatory diseases and cancer [27–29]. In our study, we found that overexpression of TGR5 inhibited the phosphorylation of IκBα and p65 translocation that induced by H/R in cardiomyocytes. These data indicated that overexpression of TGR5 effectively inhibits inflammatory response in H/R-stressed cardiomyocytes at least by inhibiting NF-κB pathway.

GSK-3β is a serine/threonine kinase, which is involved in many cellular functions including gene expression, hypertrophy and apoptosis in the heart [30,31]. GSK-3β activity is increased by phosphorylation of Tyr^216^ and decreased by Ser^9^ phosphorylation [32,33]. An important finding in the previous study indicated that inhibition of GSK-3β by phosphorylating Ser^9^ protects the heart during I/R [34]. In addition, several studies showed that phosphorylated Akt inactivates GSK-3β via Ser^9^ phosphorylation, thus decreaseing myocardial I/R injury [35,36]. AKT is a serine/threonine kinase, which plays important roles in diverse cell processes including differentiation, proliferation, survival and metabolism [37]. Kida et al. [38] reported that the TGR5 agonist (INT-777) phosphorylate AKT at Ser^473^ in aortic endothelial cells or macrophage [39]. In agreement with those reports, our results showed that overexpression of TGR5 improves phosphorylation levels of AKT and GSK-3β**.** In addition, and the specific inhibitor of AKT (MK-2206) partially blocked the effects of TGR5 on H/R-induced mitochondria dysfunction and apoptosis as well as inflammation. Together, these results support mechanistic involvement of Akt/GSK-3β signaling pathway in TGR5-mediated anti-apoptotic and anti-inflammation effects.

In summary, our results demonstrated that TGR5 offers cardioprotection against myocardial H/R injury by reducing inflammation and inhibiting mitochondria-mediated apoptosis. Activation of Akt/GSK-3β signaling is involved in the cardioprotective effect of TGR5 (Figure 5). These findings suggest that TGR5 might be an important therapeutic target for myocardial I/R injury and this finding may help in the development of a new strategy for the treatment of myocardial I/R injury.

## Supplementary Material

Supplementary Figure S1

## Abbreviations

Bcl-2: B cell lymphoma/lewkmia-2
cyto-*c*: cytochrome *c*
DMEM: Dulbecco’s Modified Eagle’s Medium
Drp1: dynamin-related protein 1
GSK-3β: Glycogen synthase kinase-3β
HCM: human cardiomyocyte
H/R: hypoxia/reoxygenation
IL: interleukin
I/R: ischemia/reperfusion
IκB: inhibitor of NF-κB
LPS: lipopolysaccharide
MMP: mitochondrial membrane potential
RIPA: Radio-Immunoprecipitation Assay
ROS: Reactive oxygen species
RT-qPCR: Quantitative Real-Time Polymerase Chain
TNF-α: tumor necrosis factor α
