# Supplementary material for: Overexpression of TGR5 alleviates myocardial ischemia/reperfusion injury via AKT/GSK-3β mediated inflammation and mitochondrial pathway
Source: Biosci Rep. 2020 Jan 24;40(1):BSR20193482. doi: 10.1042/BSR20193482 (PMC6981096; doi:10.1042/BSR20193482)
Supplement: Supplementary Figure S1 [file BSR-2019-3482_supp.pdf]

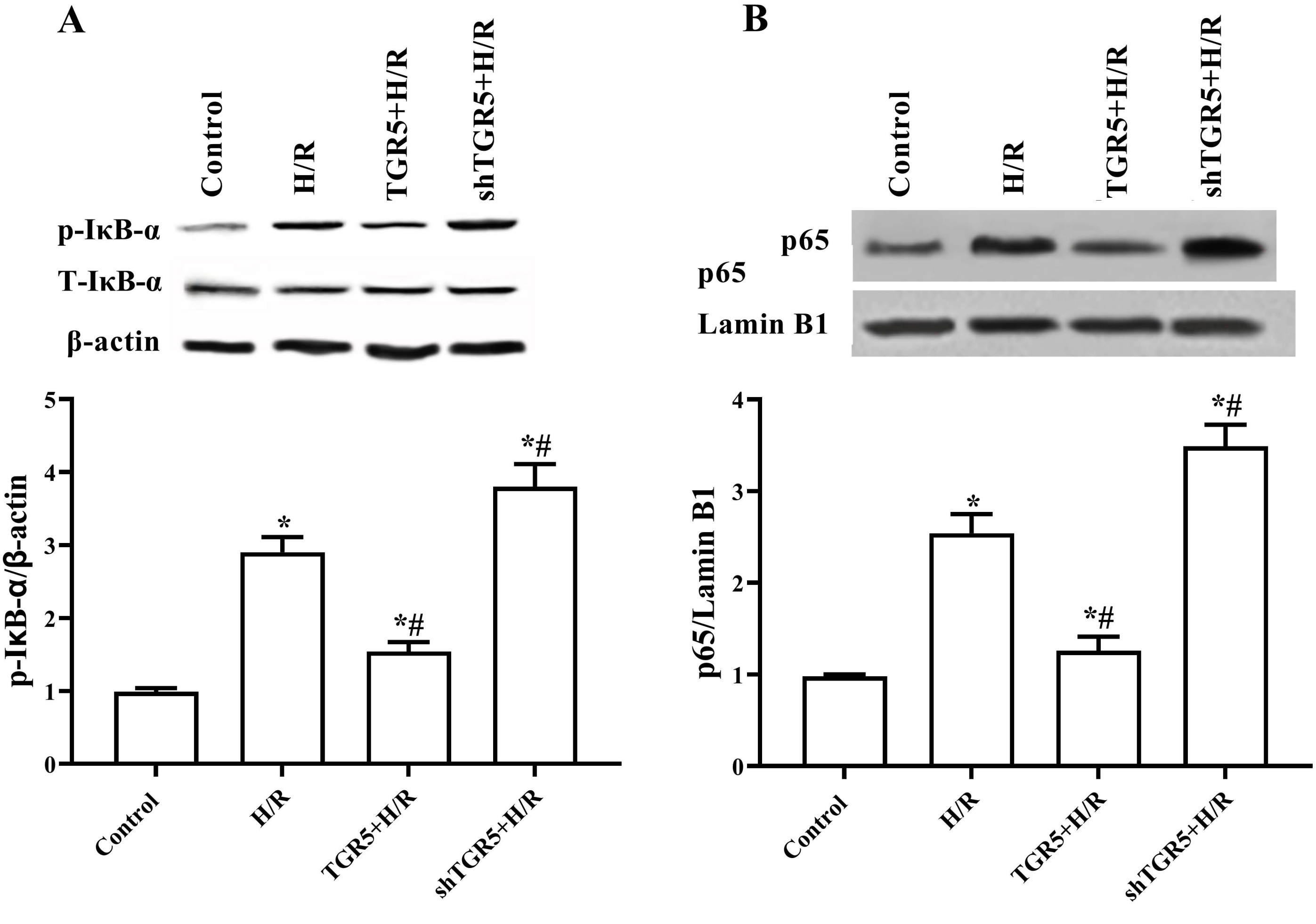

A
Control
H/R
TGR5+H/R
shTGR5+H/R
p-IκB-α
T-IκB-α
β-actin
p-IκB-α/β-actin
0
1
2
3
4
5
*
*#
*#
B
p65
p65
Lamin B1
p65/Lamin B1
0
1
2
3
4
*
*#
*#

Figure S1 overexpression of TGR5 suppressed phosphorylation of IκB-α (A) and p65 translocation (B). H9C2 cells were exposed in hypoxia condition for 4h followed by 6h of reoxygenation then transfected pcDNA3.1-TRG5 or Lentivirus-mediated TRG5-shRNAs. H/R: Hypoxia for 4h and reoxygenation for 6h; $^{*}P < 0.05$, compared with control group; $^{\#}P < 0.05$, compared with H/R group.
